# Novel Reassortant H9N2 Avian Influenza Viruses with Dual Receptor-Binding Capacity and Evidence of Direct Mammalian Infectivity Circulating in Northeast China Live Poultry Markets

**DOI:** 10.3390/v18070771

**Published:** 2026-07-13

**Authors:** Yongning Ren, Hongjin Li, Weiwen Yan, Xinxin Liu, Weiwei Chi, Rui Luo, Tobias Stoeger, Abdul Wajid, Aleksandar Dodovski, Chao Gao, Guang Wang, Maria Inge Lusida, Claro N. Mingala, Dmitry B. Andreychuk, Renfu Yin

**Affiliations:** 1 State Key Laboratory for Diagnosis and Treatment of Severe Zoonotic Infectious Diseases, Key Laboratory of Zoonosis Research, Department of Preventive Veterinary Medicine, College of Veterinary Medicine, Jilin University, Ministry of Education, Changchun 130062, China; 2 College of Food Science and Engineering, Jilin University, Changchun 130062, China; 3 Institute of Lung Health and Immunity (LHI), Comprehensive Pneumology Center (CPC), Helmholtz Zentrum München, Member of the German Center for Lung Research (DZL), 85764 Munich, Germany; 4 Department of Biotechnology, Balochistan University of Information Technology, Engineering and Management Sciences, Quetta 87300, Pakistan; 5 Department for Avian Diseases, Faculty of Veterinary Medicine, Ss. Cyril and Methodius University in Skopje, Lazar Pop Trajkov 5-7, 1000 Skopje, North Macedonia; 6 Faculty of Medicine, Universitas Airlangga, Surabaya 60115, Indonesia; 7 Research Center on Global Emerging and Re-Emerging Infectious Diseases, Institute of Tropical Disease, Universitas Airlangga, Surabaya 60115, Indonesia; 8 Livestock Biotechnology Center, Philippine Carabao Center, Science City of Muñoz, Nueva Ecija 3120, Philippines; 9 Reference Laboratory for Avian Viral Diseases, FGBI “Federal Centre for Animal Health” (FGBI “ARRIAH”), Vladimir 600901, Russia

**Keywords:** avian influenza virus, H9N2 subtype AIV, genetic evolutionary analysis, biological characteristics

## Abstract

H9N2 low-pathogenic avian influenza viruses (LPAIV) represent an ongoing zoonotic threat due to their enzootic circulation in poultry, reassortment capacity, and increasing human transmission events. This study characterized three H9N2 isolates recovered from apparently healthy poultry in a Changchun live poultry market (September–November 2022) that exhibited unprecedented genetic and phenotypic characteristics indicating enhanced zoonotic risk. Phylogenetic analysis showed a complex mosaic genome combining segments from four distinct lineages: HA from the BJ/94-like lineage (human-associated), PB1/NP/NS from the F98-like lineage, NA from the FJ/30-C-like branch, and PB2/M genes from the G1-like lineage. Bayesian molecular clock analysis estimated the most recent common ancestor at February 2022, with HL55 and HL56 diverging by May 2022, indicating rapid local viral evolution. All isolates retained hallmark LPAIV characteristics (monobasic HA cleavage site, zero intravenous pathogenicity index in chickens). However, receptor-binding assays demonstrated a critical divergence among the isolates: while HL45 exhibited exclusive avian α2-3 receptor preference, both HL55 and HL56 retained strong avian receptor binding while additionally showing measurable affinity for human α2-6 receptors-a dual-binding phenotype associated with enhanced zoonotic potential. Most significantly, the HL55 isolate successfully infected BALB/c mice without prior adaptation, causing transient upper respiratory tract replication, moderate weight loss (~9.2%), and mild disease without mortality or systemic dissemination. These findings demonstrate that the direct mammalian infectivity of this specific mosaic H9N2 lineage adds to the growing body of evidence regarding the zoonotic potential of contemporary H9N2 variants. The presence of known mammalian-adaptation markers (PB2 A588V, NA stalk deletion, HA position 226 leucine), combined with demonstrated dual receptor-binding capacity and inherent mammalian infectivity, underscores the accelerated evolutionary trajectory of H9N2 viruses toward increased zoonotic competence. These findings warrant intensified surveillance in live poultry markets, comprehensive antigenic characterization of emerging variants, and enhanced biosecurity measures to mitigate the risk of spillover events and potential pandemic emergence.

## 1. Introduction

Avian influenza viruses (AIV), members of the genus *Alphainfluenzavirus* in the family *Orthomyxoviridae*, are enveloped, segmented, negative-sense, single-stranded RNA viruses with a broad host range and notable environmental stability. These characteristics render AIV a persistent concern for both veterinary and public health. According to the World Organization for Animal Health (WOAH), AIV is categorized as highly pathogenic (HPAIV) or low pathogenic (LPAIV) based on its virulence in chickens. HPAIV, particularly H5 and H7 subtypes, causes devastating outbreaks with high mortality in poultry and occasional zoonotic transmission to humans, resulting in severe disease [1,2]. In contrast, LPAIV typically causes mild or subclinical infections in gallinaceous poultry, often leading to their under-prioritization in surveillance and control efforts. This has allowed LPAIVs to circulate enzootically in avian reservoirs, where frequent reassortment and mutation sustain their evolutionary plasticity and ongoing zoonotic risk.

Among LPAIV, H9N2 subtype viruses, first isolated in China in 1994, have become one of the most prevalent subtypes in Asian poultry, particularly in the live poultry market (LPM) [3,4,5]. Although H9N2 infections alone typically cause only mild respiratory signs in chickens, co-infections with other pathogens can exacerbate disease and lead to substantial economic losses [6,7]. Critically, H9N2 viruses frequently donate internal gene segments to emerging reassortants, including zoonotic H5Ny, H7N9, H10N8, and others [5,8]. Direct human infections with H9N2 have been documented repeatedly since the late 1990s, predominantly associated with exposure in LPM, with cases increasing in frequency in recent years [9,10]. Many contemporary Chinese H9N2 strains exhibit molecular signatures associated with mammalian adaptation, including leucine at HA position 226 (H3 numbering) in the receptor-binding site, which enhances binding to human-type α2,6-linked sialic acid receptors and contributes to dual receptor specificity [11,12].

The present study reports the isolation and comprehensive characterization of three H9N2 isolates (HL45, HL55, and HL56) from apparently healthy domestic poultry in an LPM in Changchun, Northeast China, during late 2022. These isolates display a complex mosaic genome derived from multiple lineages (BJ/94-like HA, F98-like PB1/NP/NS, FJ/30-C-like NA, and G1-like PB2/M), with high genetic similarity to both avian and certain mammalian-adapted strains. Bayesian molecular clock analysis estimated a very recent common ancestor in early 2022, reflecting rapid local evolution. All isolates retained classical LPAIV features, including a monobasic HA cleavage site and low intravenous pathogenicity index in chickens. However, receptor-binding assays revealed strong avian-type (α2-3) preference across all isolates, with HL55 and HL56 additionally exhibiting measurable human-type (α2-6) affinity. The HL55 isolate, selected for its prominent dual-binding profile, replicated transiently in the upper respiratory tract of mice without prior adaptation, causing moderate body weight loss but no mortality, systemic dissemination, or severe pathology. These results highlight the continued genetic diversification of H9N2 viruses in Chinese LPM, their acquisition of mammalian-adaptation traits, and the persistent zoonotic and pandemic risk they pose. Strengthened surveillance, antigenic monitoring, and proactive control strategies—including optimized vaccination—are urgently needed to address this evolving threat.

## 2. Materials and Methods

### 2.1. Ethical Approval

All animal experimental protocols in this study were reviewed and approved by the Experimental Animal Council of Jilin University, China, and conducted in strict accordance with institutional guidelines for animal welfare.

### 2.2. Sample Collection and Virus Isolation

Between September and November 2022, a total of 89 oropharyngeal and cloacal swabs were collected from apparently healthy domestic poultry at a live poultry market (LPM) in Changchun, Jilin province, China (longitude 125.153, latitude 43.542). Samples were transported on dry ice and stored at −80 °C until processing. The swab samples were vortexed and clarified by centrifugation at 5000× *g* for 10 min. Subsequently, the supernatants were screened for AIV RNA, and only the H9N2-positive samples were selected for inoculation into the allantoic cavities of 9- to 10- day-old SPF chicken embryos (SAIS, Jinan, China). Virus isolation in embryonated chicken embryos was performed according to the WOAH standard manual for AIV detection (WOAH Manual, Chapter 3.3.4, Version adopted in May 2023) [13]. Embryos were incubated at 37 °C for 72 h, after which allantoic fluid was harvested. Fluid lacking hemagglutination (HA) activity underwent two-blind passages; samples remaining HA-negative were considered negative for AIV. Viral subtypes and potential mixed infections were identified by RT-PCR and followed by Sanger sequencing [14].

### 2.3. RNA Extraction, RT-PCR, and Sequencing

Total RNA was extracted from infectious allantoic fluids using Trizol Reagent (Sigma, Shanghai, China), with RNA concentration and purity assessed via NanoDrop 2000 spectrophotometer (Thermo Fisher Scientific, Waltham, MA, USA). Reverse transcription was performed with the Uni12 primer (5’-AGCAAAAGCAGG-3’) using a one-step RT-PCR kit (Novoprotein, Suzhou, China). Full-genome amplification was conducted using segment-specific primers, and PCR products were separated on 1% agarose gels [14]. Positive amplicons were purified and submitted for Sanger sequencing (Kumei, Changchun, China).

### 2.4. Phylogenetic Analysis

Nucleotide sequences were assembled and edited using the Lasergene 11 software package (DNASTAR, Madison, WI, USA). Multiple sequence alignments and phylogenetic trees were constructed using the Maximum Likelihood method with 1000 bootstrap replicates in MEGA 7.0. Reference sequences were retrieved from the National Center for Biotechnology Information (NCBI) and the Global Initiative on Sharing All Influenza Data (GISAID) databases.

### 2.5. Temporal Evolutionary Analysis

A Maximum Clade Credibility (MCC) tree for the HA gene was constructed using a Bayesian Markov Chain Monte Carlo (MCMC) framework in BEAST v1.10.4 [15]. Genetic distances were analyzed via linear regression in TempEst v1.5.1. Clock and demographic models were compared using path sampling/stepping-stone sampling (PS/SSS) methods [16]. Each model was run for 10,000 iterations with 10,000,000 MCMC chains, sampled every 1000 steps. The PS/SS protocol included 50 path stages, each with 500,000 iterations. Model selection was based on log marginal likelihood estimates. Final MCMC chains were run for 500,000,000 generations, sampling every 10, 000 stages. Convergence (effective sample size >200) was confirmed using Tracer v1.6.0. The MCC tree was summarized in TreeAnnotator v1.10.4 and visualized in FigTree v1.4.4.

### 2.6. Virus Titration

Virus titers were assessed by the 50% egg infectious dose (EID_50_) method. Briefly, ten-fold serial dilutions (10^−4^ to 10^−9^) of the virus were inoculated into 10-day-old chicken embryos (five embryos per dilution). After 72 h of incubation at 37 °C, EID_50_ values were calculated using the Reed-Muench method, a statistical technique that determines the 50% infectious endpoint by accumulating the frequencies of infected and uninfected embryos across the dilution series [17].

### 2.7. Receptor-Binding Assay

Clarified infectious allantoic fluid containing the H9N2 isolates (harvested after three passages in embryonated eggs) was tested for receptor-binding assays using a solid-phase direct binding method. Viruses with HA titers ≥ 64 were adjusted to 64 HA units and pre-treated with 10 μM oseltamivir and zanamivir to inhibit neuraminidase activity. Binding to biotinylated α2-3-SA and α2-6-SA glycans coated on 96-well microtiter plates was detected using subtype-specific chicken antisera (anti-H3 and anti-H9) followed by HRP-conjugated goat anti-chicken IgG (Bioeasytech Co., Ltd. Beijing, China). Color development was performed with tetramethylbenzidine (TMB) substrate, and absorbance was measured at 450 nm.

### 2.8. Pathogenicity Test in Mice

Six- to seven-week-old female BALB/c mice were randomly divided into control and infected groups. Infected mice (*n* = 12) were anesthetized with isoflurane and intranasally inoculated with 10^6^ EID_50_ of virus in 50 µL PBS. At 3, 5, and 7 days post-inoculation (dpi), subgroups of three mice were euthanized for tissue collection (brain, nasal turbinate, trachea, lungs, spleen, and kidneys) and serum collection. Tissue viral RNA and serum hemagglutination inhibition (HI) titers were quantified in virus-inoculated mice from 3 to 14 dpi using RT-qPCR (primers, F: GACCCGAAGAAAACTGGAGGT; R: ATCCCAGTACGCACAAGAGC) and an HI assay with H9N2 antigen, respectively. Lung tissues were fixed in 4% formaldehyde for histological examination. Remaining mice were monitored daily for 14 days for body weight, clinical signs, and survival. Mice exhibiting severe clinical signs or reaching humane endpoints (≥25% body weight loss) were to be humanely euthanized immediately. To formally conclude the study, all remaining mice that survived to the pre-defined experimental endpoint at day 14 were euthanized using CO_2_ asphyxiation. This scheduled termination was an ethical requirement of the approved animal protocol, not a result of clinical deterioration.

### 2.9. Hemagglutination (HA) and Hemagglutination Inhibition (HI) Assays

Hemagglutination (HA) and hemagglutination inhibition (HI) assays were performed according to the protocols outlined in the WOAH Manual of Diagnostic Tests and Vaccines for Terrestrial Animals [13]. Briefly, for the HA assay, infectious allantoic fluids were serially diluted twofold in PBS in 96-well V-bottom microtiter plates. An equal volume of 1% (*v*/*v*) chicken red blood cells (RBCs) was added to each well. The plates were incubated at room temperature for 30 to 40 min and then visually scored for complete hemagglutination. The HA titer was defined as the reciprocal of the highest viral dilution showing complete hemagglutination.

For the HI assay, serum samples were first treated with receptor-destroying enzyme (RDE) and heat-inactivated at 56 °C for 30 min to eliminate non-specific inhibitors. The treated sera were then serially diluted twofold in PBS across 96-well V-bottom plates. Next, an equal volume of standardized H9N2 viral antigen containing 4 HA units (4 HAU) was added to each well, and the plate was incubated at room temperature for 30 min to allow for antigen–antibody binding. Following this incubation, an equal volume of 1% chicken RBCs was added to all wells. After a final incubation of 30 to 40 min at room temperature, the plates were evaluated. The HI titer was determined as the reciprocal of the highest serum dilution that completely inhibited viral hemagglutination.

### 2.10. Statistical Analysis

Data were analyzed using GraphPad Prism v11.0 (GraphPad Software Inc., San Diego, CA, USA). Differences were evaluated by one-way analysis of variance (ANOVA), with statistical significance defined as *p* < 0.05 (*) and *p* < 0.01 (**).

### 2.11. Accession Numbers

The complete genome sequences of the three H9N2 isolates have been deposited in GenBank under accession numbers OR230154 to OR230177.

## 3. Results

### 3.1. Identification and Isolation of Three H9N2 Subtype AIV Isolates from Apparently Healthy Domestic Poultry in Live Poultry Markets in Northeastern China

Between September and November 2022, a total of 89 oropharyngeal and cloacal swabs were collected from apparently healthy domestic poultry at a live poultry market in Changchun, Jilin province, Northeast China (longitude 125.153, latitude 43.542). Using AIV-specific primers in combination with RT-PCR, Sanger sequencing, and BLAST (https://blast.ncbi.nlm.nih.gov/Blast.cgi, accessed on 8 July 2026) homology searches, three H9N2 subtype AIV isolates were identified, and no other influenza A subtypes were detected in any of the three samples. Newcastle disease virus (NDV) and other avian avulaviruses were also excluded by RT-PCR (primer sequences are available from the corresponding author upon request). H9N2 RNA-positive samples were inoculated into the allantoic cavities of 9- to 10-day-old SPF chicken embryos following WOAH standard protocols for AIV detection. All three isolates were successfully propagated, with hemagglutinin (HA) titers ranging from 2^5^ to 2^7^ (32–128 reciprocal dilutions) in the harvested allantoic fluid. The isolates were designated A/chicken/Jilin/HL45/2022 (HL45), A/pigeon/Jilin/HL55/2022 (HL55), and A/pigeon/Jilin/HL56/2022 (HL56). Viral titers, determined by EID_50_ assay, ranged from 10^6.361^ to 10^5.72^ EID_50_/mL. The complete genomes of the three isolates were obtained by RT-PCR amplification and followed by Sanger sequencing [14]. Following RT-PCR amplification, Sanger sequencing confirmed that the complete viral genomes, comprising all eight intact gene segments rather than genomic fragments, were successfully obtained for the three isolates. The complete genome sequences of the three isolates have been deposited in GenBank under accession numbers OR230154 to OR230177. In conclusion, three H9N2 subtype isolates (HL45, HL55, and HL56) were successfully identified and isolated from apparently healthy domestic poultry at a live poultry market in Changchun, Northeast China, in late 2022.

### 3.2. The Three H9N2 Subtype AIV Isolates Exhibit Complex Evolutionary Patterns Involving Multiple Lineages

**Figure 1:**
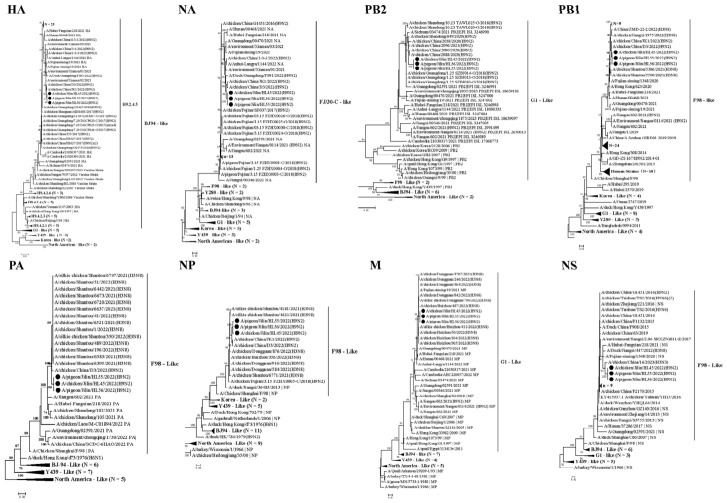
Maximum likelihood phylogenetic trees of the eight viral gene segments of three H9N2 AIV isolated from domestic poultry in a live poultry market in Changchun, Jilin Province (September–November 2022), with selected reference sequences. Trees were constructed in MEGA 7.0 using the Maximum Composite Likelihood model and 1000 bootstrap replicates. Bootstrap values ≥70% are shown at major nodes. Isolates from this study are marked with solid circles.

To gain deeper insights into the evolutionary origins of the three H9N2 subtype AIV isolates described in this study, we conducted a comprehensive phylogenetic analysis of all eight viral gene segments. This analysis incorporated the three isolates and a diverse set of representative reference viruses retrieved from GenBank. Phylogenetic reconstruction revealed that the three isolates displayed four distinct evolutionary patterns across their gene segments: the HA gene clustered within the BJ/94-like lineage; the PB1, NP, and NS genes grouped with the F98-like lineage; the NA gene belonged to the FJ/30-C-like branch; and the PB2 and M genes clustered with the G1-like lineage (Figure 1) [10,18,19,20,21].

Nucleotide sequence comparisons further indicated that the three H9N2 isolates shared the highest overall similarity with other avian-origin H9N2 viruses (GenBank accession numbers OQ130591.1, PV375662.1, ON368080.1, OR528484.1, OQ293196.1, PV375121.1, PV376481.1, and PV377464.1). Nevertheless, certain gene segments, including HA, PA, M, and NS, also showed high nucleotide identity (≥98%) with corresponding genes from H3N8 AIV and H9N2 isolates from non-avian species (Table 1). This finding suggests possible historical gene exchange or shared ancestry between avian and mammalian-adapted strains.

At the molecular level, a notable three-amino-acid deletion (positions 62–64 in the stalk region) was identified in the NA protein of all three isolates. This deletion is a well-recognized molecular signature associated with the adaptation of H9N2 viruses from aquatic birds to terrestrial poultry [22,23]. In addition, several amino acid substitutions previously linked to enhanced viral pathogenicity were identified, including NS1 V149A (associated with enhanced viral dissemination in chickens), PB2 V147I (linked to increased pathogenicity in mice), and PB2 A588V (reported to improve replication efficiency in both avian and mammalian hosts [21,24] (Table 2). Collectively, these genetic and molecular characteristics underscore the remarkable interspecies adaptability of contemporary H9N2 viruses and their continued potential for cross-species transmission, highlighting the critical need for sustained surveillance [25].

**Figure 2:**
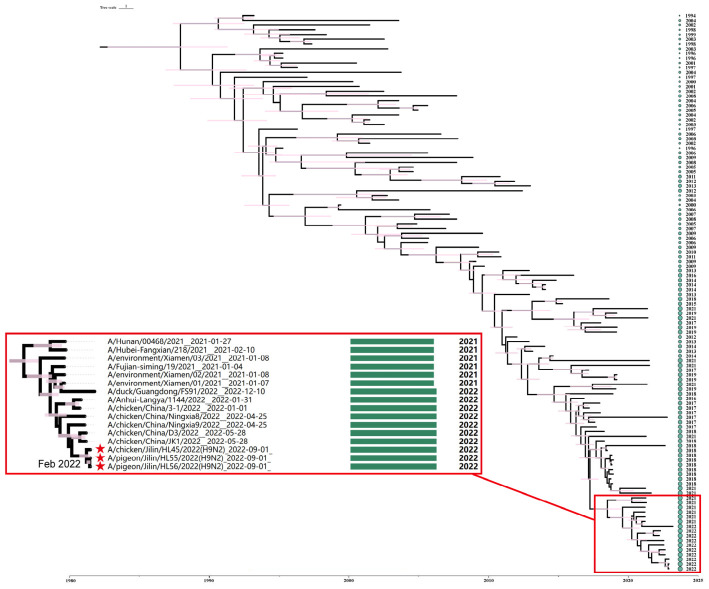
Bayesian time-resolved phylogenetic tree based on the complete HA gene segments of three H9N2 AIV isolates and reference sequences. The estimated time to the most recent common ancestor (tMRCA) for the newly isolated clade is explicitly indicated at the designated node (e.g., February 2022). Purple shading represents the 95% highest posterior density (HPD) intervals for node ages. Isolates from this study are marked with red five-pointed star.

To further elucidate the temporal dynamics of these H9N2 viruses, a Bayesian time-resolved phylogenetic tree was constructed using the HA gene sequences (Figure 2). The analysis revealed a strong temporal signal, with the time to the most recent common ancestor (tMRCA) of the three isolates estimated to be February 2022 (95% highest posterior density [HPD] interval: January–March 2022). Among the three isolates, the HL45 isolate diverged earliest, with an inferred origin in early February 2022, followed by the subsequent divergence of HL55 and HL56 around May 2022 (95% HPD interval: April–June 2022).

In summary, the three H9N2 isolates possess complex mosaic genome structures derived from multiple established lineages and exhibit high genetic similarity to both avian and certain mammalian-adapted strains in several gene segments. The presence of characteristic molecular markers, including the NA stalk deletion and mammalian-associated PB2 mutations, indicates ongoing adaptation potential. Bayesian molecular clock analysis further supports a very recent common ancestor in early 2022, underscoring the rapid evolutionary dynamics of H9N2 viruses in the region and reinforcing the need for continuous and enhanced surveillance to monitor genetic changes and assess the risk of zoonotic transmission.

### 3.3. Molecular Characterization and Pathogenicity Assessment Confirm the Low Pathogenic Nature of the Three H9N2 Isolates in Chickens

To assess the virulence of the three H9N2 isolates, the HA protein cleavage site motif and the intravenous pathogenicity index (IVPI) were determined in accordance with the WOAH standards for avian influenza (including infection with high pathogenicity avian influenza viruses) detection. The HA cleavage site motif of all isolates was PSKSSR↓G, characterized by a single basic amino acid and the absence of multiple consecutive basic residues or insertions—a hallmark of LPAIV. In 4-week-old SPF chickens, the IVPI was 0.00, and no clinical signs were observed throughout the observation period. Additionally, the mean death time (MDT) in 9- to 10-day-old SPF embryonated chicken eggs exceeded 168 h, with no embryo mortality occurring by 7 dpi. Collectively, these molecular and pathogenicity data demonstrate that all three H9N2 isolates exhibit low pathogenicity in chickens.

### 3.4. Diverse Receptor-Binding Profiles of the Three H9N2 Isolates to Avian- and Human- Derived Receptors

**Figure 3:**
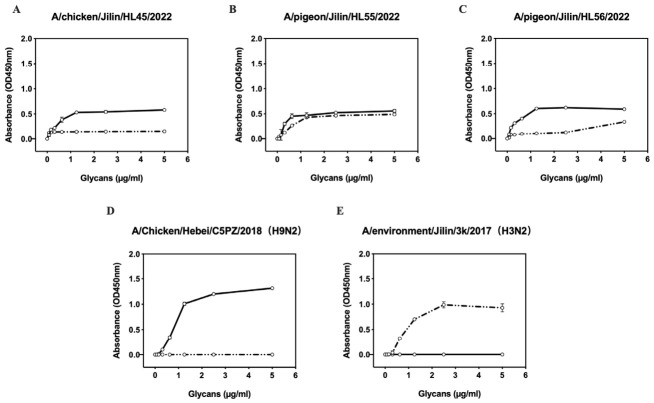
Receptor-binding properties of the H9N2 AIV isolates. Binding affinities to avian-type (α2-3-linked sialic acid, α2-3-SA) and human-type (α2-6-linked sialic acid, α2-6-SA) receptors were assessed using a solid-phase direct binding assay with synthetic trisaccharide receptors. HL44, HL45, and HL56 strains (**A**–**C**) showed varying binding affinities to both α2-3-SA and α2-6-SA receptors, with the dotted and solid lines denoting binding to human- and avian-type receptors, respectively. The dotted and solid lines denote binding to human- and avian-type receptors, respectively. The control strains A/Environment/Jilin/3k/2017 (H3N2) and A/Chicken/Hebei/C5PZ/2018 (H9N2) displayed high affinities for α2-6-SA and α2-3-SA, respectively, as indicated by the solid and dotted lines (**D**,**E**). Data represent mean values from two independent experiments with consistent results.

The receptor-binding preferences of the three H9N2 isolates for avian-derived (α2-3-SA) and human-derived (α2-6-SA) receptors were assessed using solid-phase receptor-binding assays (Figure 3). The control strains A/Environment/Jilin/3k/2017 (H3N2) and A/Chicken/Hebei/C5PZ/2018 (H9N2) displayed high affinities for α2-6-SA and α2-3-SA, respectively, as indicated by the blue and red lines (Figure 3D,E). All three H9N2 isolates exhibited strong binding to α2-3-SA, reflecting a predominant preference for avian-type receptors (Figure 3A–C). However, notable differences were observed in their binding to human-type receptors. The HL45 isolate bound exclusively to α2-3-SA and showed no detectable affinity for α2-6-SA. In contrast, HL55 and HL56 retained strong α2-3-SA binding while also exhibiting measurable affinities for α2-6-SA, suggesting an increased potential for cross-species transmission to humans. Notably, among the three isolates, HL55 exhibited the highest binding capacity for α2-6-SA (Figure 3B), further highlighting its zoonotic risk. Collectively, these findings reveal substantial diversity in receptor-binding profiles among the three H9N2 isolates and underscore the importance of continued surveillance for strains exhibiting enhanced binding to human receptor-type receptors. Based on its receptor-binding affinity characteristics, HL55 was selected for subsequent mammalian infection studies to further assess its cross-species transmission potential.

### 3.5. Infection with the HL55 Isolate Causes Transient Upper Respiratory Tract Infection and Moderate Body Weight Loss in Mice Without Prior Adaptation

**Figure 4:**
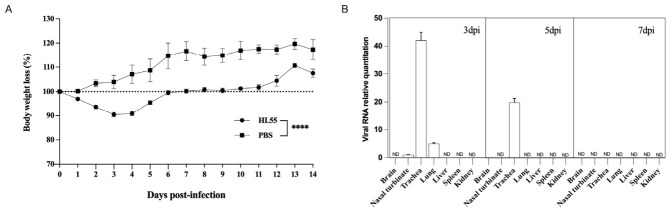
Pathogenicity of the H9N2 HL55 isolate in mice. Groups of five 6-week-old BALB/C mice were intranasally inoculated with 10^6^ EID50 of the HL55 isolate in 50 μL volume. Body weight and survival were monitored daily for 14 dpi. Mice exhibiting ≥25% body weight loss were humanely euthanized. (**A**) Percent change in body weight following infection. (Data are presented as mean ± SEM from three independent experiments. **** *p* < 0.0001 vs. the PBS group.) (**B**) Viral RNA loads in selected tissues at 3, 5 dpi and 7 dpi, quantified by RT-qPCR.

To assess the pathogenicity of the H9N2 isolate HL55 in a mammalian model, six-week-old female BALB/c mice were intranasally inoculated with 10^6^ EID_50_ of the virus in a 50 μL volume and monitored for 14 dpi. No overt clinical signs were observed, and all challenged mice survived through the experiment (100% survival rate). The primary clinical manifestation was transient body weight loss, which began at 1 dpi and peaked at 9.2% reduction by 3 dpi (Figure 4A). Body weight subsequently recovered gradually but remained below that of the PBS-inoculated control group throughout the observation period. Viral loads in multiple tissues, including the brain, nasal turbinate, trachea, lungs, liver, spleen, and kidneys, were quantified by RT-qPCR. Viral RNA was not detected in the liver, spleen, kidney, or brain at any time point during the observation period. In contrast, viral RNA was transiently detected in respiratory tissues at 3 and 5 dpi, but not at 7 dpi. Specifically, viral RNA was detected in the nasal turbinate at 3 dpi and in both the trachea and lungs at 3 and 5 dpi. Histopathological examination of lung tissues showed no obvious lesions in infected mice from 3 to 7 dpi, despite the presence of viral RNA in the respiratory tract. However, viral RNA was completely cleared and undetectable in all collected tissues from 7 dpi through 14 dpi. Furthermore, serum HI titers from 3 dpi to 14 dpi remained undetectable. Among the respiratory tissues, the trachea consistently exhibited the highest viral loads, indicating a predominant upper respiratory tract infection. Collectively, these results indicate that while the HL55 isolate is capable of short-term replication within the respiratory system without prior adaptation (Figure 4B), the infection is self-limiting and fails to elicit a robust systemic antibody response. Ultimately, the HL55 isolate can directly infect mice without prior adaptation, resulting in transient upper respiratory tract infection and moderate body weight loss, while causing only mild disease overall in this murine model.

## 4. Discussion

This present study describes the isolation and comprehensive characterization of three H9N2 subtype isolates (HL45, HL55, and HL56) from apparently healthy domestic poultry at an LPM in Changchun, Northeast China, during late 2022. The complex mosaic genomic structure of these isolates, featuring HA from the BJ/94-like lineage, PB1/NP/NS from F98-like, NA from FJ/30-C-like, and PB2/M from G1-like lineages, aligns closely with the predominant genotypes circulating in Chinese poultry over the past decade, particularly the widespread G57 (genotype S) lineage that has dominated since around 2013 (Figure 1).

Phylogenetic and nucleotide identity analyses revealed complex evolutionary dynamics, with evidence of genetic exchange not only among avian-origin H9N2 strains but also between certain segments (HA, PA, M, and NS) that exhibited high similarity (≥98%) to H3N8 AIV and H9N2 viruses from non-avian hosts (Table 1). This inter-lineage and potential interspecies genetic exchange highlights the ongoing risk of reassortment events in LPM settings, which serve as critical mixing vessels for influenza A viruses and may facilitate the emergence of variants with increased zoonotic potential [5,9,10,11,26,27].

Bayesian molecular clock analysis of the HA gene estimated the time to the most recent common ancestor (tMRCA) of the three isolates in February 2022 (95% HPD: January–March 2022), with subsequent divergence of HL55 and HL56 occurring around May 2022. These findings indicate remarkably recent local evolution, likely driven by frequent viral introductions and sustained transmission within the dynamic LPM environment, consistent with reports of rapid intra-market evolutionary turnover in contemporary H9N2 populations [11,28,29].

All three isolates exhibited classical molecular signatures of low-pathogenic AIV (LPAIV), including monobasic HA cleavage site motifs (PSKSSR↓G), an IVPI of 0.00 in SPF chickens, and MDT > 168 h in embryonated eggs. These features are typical of the vast majority of currently circulating H9N2 viruses in gallinaceous poultry, which rarely induce overt clinical disease yet persist enzootically through subclinical infections [30,31]. It should be noted that only genetic material (RNA) was obtained from the original swabs via RT-PCR and Sanger sequencing, and complete viral genomes were recovered for all three isolates. While this procedure satisfies WOAH requirements for AIV detection, it provides circumstantial evidence for virus isolation as defined by classical criteria, and the reisolation of viral particles via filtration and centrifugation was not attempted. Electron microscopy characterization was not performed in this study. Despite the demonstrated ability to infect mammalian cells and cause mild disease in mice, the low pathogenic potential of these isolates in chickens (IVPI = 0.00) reflects their LPAIV nature. This is consistent with their genetic signatures and receptor-binding profiles, which suggest enhanced zoonotic potential without the strong pathogenicity associated with HPAIV.

Receptor-binding assays demonstrated a conserved strong preference for avian-type receptor preference (α2-3-SA) across all isolates, consistent with their avian origin and adaptation to terrestrial poultry. Notably, however, HL55 and HL56 also exhibited measurable binding affinity for human-type receptors (α2-6-SA), whereas HL45 showed exclusive α2-3-SA specificity. Dual receptor-binding phenotypes have been increasingly documented among contemporary poultry-adapted H9N2 strains and represent an important molecular indicator of elevated zoonotic potential [10,27,32]. In line with this observation, the HL55 isolate—which exhibited the strongest α2-6-linked receptor binding—successfully infected BALB/c mice without prior adaptation, achieving transient replication in upper respiratory tissues (nasal turbinate, trachea, and lungs) and causing moderate, self-limiting weight loss (peak ~9.2% at 3 dpi) without mortality or systemic spread changes in the lung tissue. These results demonstrate that certain contemporary H9N2 variants possess inherent capacity for mammalian infection and mild pathogenesis, reinforcing their potential role as progenitors or contributors to future zoonotic spillover events. Future studies should include attempts at virus reisolation from infected tissues using cell culture or embryonated eggs, electron microscopy characterization of viral particles, and verification of infectivity through contact transmission experiments. These additional experiments would strengthen the evidence for active viral replication and transmission potential.

While our data demonstrate that the three isolates retain hallmark low-pathogenic avian influenza virus (LPAIV) characteristics in chickens—including a monobasic HA cleavage motif, an IVPI of 0.00, and a prolonged MDT in embryonated eggs—the low pathogenic potential observed in the murine model must be interpreted carefully. The transient weight loss and absence of severe pathology in mice suggest limited respiratory infectivity rather than high virulence in mammals. Crucially, our evaluation of viral replication in mice relied exclusively on the quantification of viral RNA via RT-qPCR. While this provides strong genetic evidence of infection, it does not directly confirm the production or shedding of live, intact virions. Therefore, further experiments are required to verify the existence of specific infectious viral particles corresponding to the detected genetic material. Future studies should incorporate virus re-isolation from infected tissues (using embryonated eggs or permissive cell cultures), morphological confirmation via electron microscopy or other particle-level characterizations, and, where appropriate, contact-transmission experiments. These methodologies will be essential to definitively link the detected genetic material to infectious virions and fully elucidate the zoonotic and transmission potential of these emerging variants.

Finally, we must note a methodological limitation regarding the definition of virus isolation. While our virus detection and propagation protocols strictly followed WOAH diagnostic standards—relying on specific pathogen-free (SPF) embryonated egg inoculation, hemagglutination assays, and whole-genome sequencing—this approach provides circumstantial and genetic evidence of the virus. In accordance with Rivers’ criteria, true virus particle isolation requires the purification and direct morphological confirmation of the infectious agent, typically validated by electron microscopy, which was not performed in this study [33]. Likewise, the evaluation of viral replication in our murine model was exclusively determined by the quantification of viral RNA using RT-qPCR. The reisolation of infectious viral particles from the mouse tissues was not attempted. Therefore, while our data comprehensively characterize the genetic and functional attributes of these H9N2 variants, future studies incorporating electron microscopy and live-virus reisolation from hosts are warranted to provide direct physical evidence of the infectious virions.

In summary, this study underscores the ongoing genetic diversification and mosaic evolution of H9N2 viruses in Chinese LPMs, where select isolates have acquired molecular determinants associated with mammalian receptor preference and limited mammalian infectivity. Given the continued dominance of such genotypes in poultry populations and the demonstrated direct mouse infectivity of HL55 without adaptation, these findings emphasize the critical need for enhanced surveillance vigilance. Strengthened monitoring in LPMs, coupled with systematic antigenic and functional characterization of emerging variants, is essential to detect early indicators of increased zoonotic or pandemic risk and inform timely implementation of control measures.

## Figures and Tables

**Table 1:** Amino acid sequence identity of the eight gene segments from the three H9N2 isolates (HL45, HL55, and HL56) compared with the top five reference strains exhibiting the highest identity retrieved from the GenBank database. Because the three isolates are highly homologous, they share the same top reference strains, and the values represent the identity range across all three isolates. The different lineages of the H9N2 subtype are distinguished by color, with BJ94-like, J/30-C-like, G1-like, and F98-like lineages represented in light green, dark green, yellow, and orange, respectively. Non-H9N2 reference strains (H3N8) are uncolored.

Gene	Virus with Highest Identity	Subtype (Lineage)	Identity with HL45, HL55, and HL56 (%)	GenBank Accession No.
HA	A/chicken/China/def3,2022BC/2022(H9N2)	H9N2 (BJ94-like)	99.23–99.41	OQ130591.1
	A/chicken/China/D3/2022(H9N2)		99.11–99.29	OR053999.1
	A/chicken/China/JK1/2022(H9N2)		99.05–99.23	OR054001.1
	A/Fujiansiming/19/2021(H9N2)		98.93–99.11	ON856627.1
	A/duck/Hunan/01.26_YYWLP-C14/2022(H9N2)		99.05–99.11	PV374024.1
NA	A/chicken/Fujian/6.29FZHX0710-178-2-C/2021(H9N2)	H9N2 (FJ/30-C-like)	99.36	PV375662.1
	A/chicken/Fujian/2.04FZHX0710-95-2-O/2021(H9N2)		99.29	PV375664.1
	A/duck/Hunan/01.26_YYWLP-C14/2022(H9N2)		99.29	PV379738.1
	A/chicken/Shandong/12.30TAFC16-O/2021(H9N2)		99.00	PV375548.1
	A/chicken/Shanxi/6-9JZRL0011-O/2022(H9N2)		98.93	PV381362.1
PB2	A/chicken/China/2088/2020(H9N2)	H9N2 (G1-like)	98.15–98.24	ON368080.1
	A/chicken/China/2096/2021(H9N2)		98.11–98.2	ON376799.1
	A/chicken/Shanxi/10.26_JZRL10-O/2020(H9N2)		98.07–98.16	PV381189.1
	A/chicken/Shandong/049/2020(H9N2)		98.07–98.16	MZ703045.1
	A/chicken/Guangdong/1.25_SZBJ013-O/2018(H9N2)		97.98–98.11	MW103549.1
PB1	A/chicken/China/D3/2022(H9N2)	H9N2 (F98-like)	99.38–99.43	OR528484.1
	A/chicken/China/JK1/2022(H9N2)		99.38–99.43	OR528486.1
	A/chicken/Fujian/2.04FZHX0710-95-2-O/2021(H9N2)		99.25–99.30	PV372270.1
	A/chicken/Shandong/4.30TAFC25-O/2022(H9N2)		99.16–99.21	PV381006.1
	A/chicken/Huizhou/2524/2021(H3N8)	H3N8	98.72–98.77	OQ292067.1
PA	A/chicken/Shantou/6521/2021(H3N8)	H3N8	98.93–99.07	OQ293196.1
	A/chicken/Fujian/2.04FZHX0710-95-2-O/2021(H9N2)	H9N2 (F98-like)	98.93–99.07	PV373005.1
	A/chicken/Shandong/4.30TAFC25-O/2022(H9N2)		98.93–99.07	PV381007.1
	A/chicken/Shantou/1/2022(H3N8)	H3N8	98.88–99.02	OQ292708.1
	A/chicken/Shantou/6637/2021(H3N8)		98.88–99.02	OQ293228.1
NP	A/chicken/Shanxi/6-9JZRL0005-O/2022(H9N2)	H9N2 (F98-like)	99–99.13	PV375121.1
	A/chicken/Shanxi/7-28JZRL0065-O/2022(H9N2)		98.93–99.06	PV375152.1
	A/chicken/Fujian/6.29FZHX0710-178-2-C/2021(H9N2)		98.86–99	PV374902.1
	A/silkie chicken/Shantou/4181/2021(H3N8)	H3N8	98.26–98.4	OQ293622.1
	A/silkie chicken/Shantou/4621/2021(H3N8)		98.2–98.36	OQ293670.1
M	A/chicken/Shandong/12.30TAFC7-O/2021(H9N2)	H9N2 (G1-like)	99.39	PV376481.1
	A/chicken/Henan/12.29WJX8-C/2021(H9N2)		99.29	PV376429.1
	A/chicken/Dongguan/364/2022(H3N8)	H3N8	99.19	OQ291664.1
	A/chicken/Dongguan/842/2022(H3N8)		99.08	OQ291840.1
	A/chicken/Huizhou/104/2022(H3N8)		98.98	OQ291992.1
NS	A/chicken/Shandong/12.30TAFC19-O/2021(H9N2)	H9N2 (F98-like)	99.16	PV377464.1
	A/duck/Hunan/01.26_YYWLP-C14/2022(H9N2)		99.16	PV377757.1
	A/chicken/Fujian/6.29FZHX0710-178-2-C/2021(H9N2)		99.05	PV377620.1
	A/chicken/Fujian/2.04FZHX0710-95-2-O/2021(H9N2)		98.93	PV377622.1
	A/chicken/Shantou/6799/2021(H3N8)	H3N8	98.45	OQ293385.1

**Table 2:** Key amino acid sites associated with receptor-binding specificity, pathogenicity, and host adaptation in the HA, NA, PB2, M2, and NS1 proteins of the three H9N2 isolates compared with selected reference strains. Note: Amino acid positions in the HA protein are numbered according to the mature H3 hemagglutinin numbering system to ensure consistent alignment. The “↓” denotes the cleavage site.

Virus Strain	HA Protein (H3 Numbering)		NA Protein	PB2 Protein	M2	NS1
	RBS Positions (158, 183, 189, 190, 226, 227, 228)	Cleavage Site (333–340)	Stalk Deletion (62–64)	Mutations (147, 627, 701, 588)	S31N	V149A
A/Duck/HongKong/Y439/1997(H9N2)	S, H, T, E, Q, Q, G	PSRSSR↓G	YES	I, E, D, A	S	A
A/Quail/HongKong/G1/1997 (H9N2)	S, H, T, E, L, Q, G	PSRSSR↓G	YES	M, E, D, A	S	A
A/Chicken/Shanghai/F/1998(H9N2)	N, N, T, A, Q, Q, G	PSRSSR↓G	NO	I, E, D, A	N	A
A/Chicken/Beijing/1/1994 (H9N2)	N, N, T, V, Q, Q, G	PSRSSR↓G	YES	V, E, D, A	S	A
A/Duck/HongKong/Y280/1997(H9N2)	N, N, T, T, L, Q, G	PSRSSR↓G	NO	V, E, D, A	S	A
A/Chicken/Shanghai/06/2015(H9N2)	N, N, D, T, L, M, G	PSRSSR↓G	NO	I, E, D, A	N	A
A/Chicken/Fujian/C1161/2013(H9N2)	N, N, T, A, L, Q, G	PSRSSR↓G	NO	I, E, D, A	N	A
A/HongKong/33982/2009(H9N2)	S, H, T, D, Q, Q, G	PSRSSR↓G	YES	M, E, N, A	S	A
A/Fujiansiming/19/2021(H9N2)	D, N, T, A, Q, Q, G	PARSSR↓G	YES	-, -, -, -	-	-
A/Chicken/Jilin/HL45/2022(H9N2)	N, N, D, V, L, M, G	PSKSSR↓G	YES	I, E, D, V	N	A
A/Pigeon/Jilin/HL55/2022(H9N2)						
A/Pigeon/Jilin/HL56/2022(H9N2)						

## Data Availability

The raw data supporting the conclusions of this article will be made available by the authors on request.
